# Qualitative validation of TnED©, an electronic instrument capturing pain dimensions in patients with trigeminal neuralgia

**DOI:** 10.22514/jofph.2026.038

**Published:** 2026-05-12

**Authors:** Amine S. Tahiri, Serena A. Dodhia, Sneha Chotaliya, Hossein Ansari, George Garibaldi, Robert Lasser, Joanna M. Zakrzewska

**Affiliations:** ^1^Noema Pharma AG, 4051 Basel, BS, Switzerland; ^2^King’s College Hospital, SE5 9RS London, UK; ^3^Kaizen Brain Centre, San Diego, CA 92037, USA; ^4^Department of Neurology, University of California San Diego (UCSD), San Diego, CA 92121, USA; ^5^Pain Management Centre, Eastman Dental Institute, University College London, WC1E 6DG London, UK

**Keywords:** Trigeminal neuralgia, Validation, Pain, Qualitative, Patient reported outcomes, Content validity

## Abstract

**Background**: Trigeminal neuralgia (TN) is a severe episodic facial pain that has a significant impact on mood and activities of daily living. To accurately measure pain in TN, patient diaries need to be specifically developed to capture dimensions of pain in both TN phenotypes (*e.g.*, purely paroxysmal *vs.* paroxysms with concomitant continuous pain) and their impact on daily life. There is no validated pain instrument that measures specific symptoms of the pain associated with TN. Therefore, Noema Pharma decided to initiate the development and validation of such an instrument. The purpose of this study was to evaluate the content validity of the items that make up the Trigeminal Neuralgia Electronic Diary (TnED©). **Methods**: Participants were divided into two groups: those with purely paroxysmal pain and those with paroxysmal and concomitant continuous pain; each group was provided with a data collection instrument (diary) specific to their TN phenotype. Participants completed the instrument daily for 14 days. A qualitative, semi-structured interview was conducted to collect feedback on the instrument. **Results**: A total of 30 participants were enrolled in 2 English-speaking countries (USA, UK). Nearly all participants stated that the items were clear and easy to understand, relevant, measured all symptoms of the condition, and had a suitable recall period and response options. Some participants suggested additional items to be collected. Most participants regarded pain severity as the most important concept due to its debilitating impact on their lives. Few changes to the instrument were suggested, and the study sponsor decided not to implement any of the proposed changes. **Conclusions**: Overall, the Trigeminal Neuralgia Electronic Diary was well-received and can be used to measure symptoms of TN in clinical trials or in a clinical setting. Psychometric validation of the TnED© instrument is ongoing. **Clinical Trial Registration**: NCT06019338, retrospectively registered.

## 1. Introduction

Trigeminal neuralgia (TN) is a severe, orphan facial pain condition [1, 2] and is defined by the International Classification of Orofacial Pain as “recurrent unilateral brief electric shock-like pain, abrupt in onset and termination, limited to the distribution of one or more divisions of the trigeminal nerve” [3]. It is usually triggered by innocuous stimuli, touch, or normal activities, such as talking and shaving. There may also be concomitant continuous pain of variable intensity within the distribution(s) of the affected nerve division(s); this concomitant continuous pain is often described as dull or throbbing and can last several days [3]. TN pain’s severity and impact on activities of daily living (ADLs) result in significant burden, including depression, anxiety, and potentially suicide [4, 5].

TN is generally classified into three types (classical, secondary, and idiopathic) with two identifiable phenotypes: purely paroxysmal TN (TNP) and TN with concomitant continuous pain (TNC) [6].

Measuring the pain associated with TN is made difficult by the considerable number of heterogeneous and often discordant questionnaires being used in TN studies, the few efforts to validate existing questionnaires, and the suboptimal evidence uncovered in such validation attempts [7, 8, 9]. To date, there is no validated data collection instrument (diary) specifically adapted to the symptoms of TN. Thus, there is an urgent need to develop psychometrically sound patient-reported outcome measures (PROMs) to assess TN.

Paper diaries are frequently used in TN clinical trials to assess a patient’s condition (*e.g.*, symptom severity, quality of life (QoL)) or to measure treatment compliance. Frequent recording of symptoms using a diary helps to reduce recall bias. To accurately measure pain in patients with TN, the instrument needs to be specifically developed to capture the different dimensions of pain in each TN phenotype (*i.e.*, TNP *vs.* TNC), have the shortest recall period possible, and not allow retrospective editing [10]. Additionally, the instrument should have good content validity: it should be relevant for the context of interest (within a specific population and context of use); it should be comprehensive with respect to patient concerns; and its instructions, items, response options, and recall period should be understood by the population of interest as intended [11]. The validation of diary instruments and other PROMs in TN studies should be a priority in this field of research [8].

Noema Pharma is developing basimglurant, a potential first-in-class investigational negative allosteric modulator of the metabotropic glutamate 5 receptor, for the treatment of TN and is currently conducting a Phase 2/3 clinical trial (NOE-TGN-201 Libra-TN as reported on clinicaltrials.gov site; identifier: NCT05217628). One outcome measure in this clinical trial is the Trigeminal Neuralgia Electronic Diary (TnED©), which was developed by a multidisciplinary team with input from patient-reported outcome specialists, statistical experts, and clinicians experienced in managing TN patients, as well as TN patient associations. In line with US Food and Drug Administration guidelines, the first stage in the development of a PROM is to ensure concepts important to patients are captured and understood by the patients with suitable response options and recall periods (content validity) [11].

This study aimed to explore the content validity of the items that make up the TnED© in patients who suffer from TNP or TNC in the UK and USA.

## 2. Materials and methods

### 2.1 Ethical considerations

Two patient-members of the Facial Pain Association provided input on the development of the TnED©. This study was conducted in compliance with Good Clinical Practice, including International Council for Harmonisation guidelines. In addition, all applicable local laws and regulatory requirements were adhered to throughout the study.

### 2.2 Research team

The UK research team consisted of one supervising leading TN specialist (medical doctor and dentist) and two dentist researchers. The two dentist researchers were responsible for recruiting participants and facilitating the semi-structured interviews.

The US research team consisted of one TN disease expert assisted by a leading facial pain specialist who was responsible for recruiting participants, and a study coordinator who was responsible for the semi-structured interviews.

Analysis of the collected data was performed by experts from an independent vendor (Clinical Outcomes Solution, UK) specialized in PROM validation.

### 2.3 Recruitment and sample size

The study was conducted in La Jolla, California, US, and in London, UK, with participants who met the study inclusion criteria. A sample size of up to 20 participants was estimated sufficient to meet the study objectives, since saturation is typically reached within 10–15 interviews [12]. Participants were compensated for their time and travel costs. No formal criteria on diversity of the enrolled population were pre-specified, due to the low prevalence of TN. Investigators partnered with the patients’ associations in the US and the UK to inform as many patients as possible of the study and maximize the likelihood of recruiting a representative sample.

Participants were of any gender, 18 years of age and over, with current or former clinically diagnosed TN. Additional inclusion criteria were access to a smartphone or tablet, good command of the English language, commitment to fill in the diary according to the instructions, and willingness to take part in a virtual interview after completion of the study instrument. Participants were eligible to participate regardless of their medical history.

### 2.4 Data collection

The TnED© was developed as an app (TrialOnline™, Replior AB, Stockholm, Sweden). No therapeutic intervention or treatment was administered as part of this non-interventional study.

TnED© data collection and interviews were conducted between November 2021 and August 2023.

#### 2.4.1 TnED© completion

Based on their TN phenotype, participants were allocated to either Diary 1 (TNP) or Diary 2 (TNC). The instrument was completed electronically on a smartphone App (using the participants’ own phones) every evening for 14 days. Participants were provided with an instruction document to support with the set-up and use of the TnED© App.

#### 2.4.2 TnED© description

The TnED© is a PROM that contains several patient-reported items grouped to make a disease-specific and phenotype-specific instrument. Both phenotype instruments contained the following 3 items: number of pain attacks split by day sub-periods (morning, midday, evening, night) in the past 24 hours; pain attack severity (scale of 0 to 10), and pain interference with daily activities (scale of 0 to 10). For participants with TNC, the instrument contained two additional items: continuous pain duration in the past 24 hours; and continuous pain severity (scale of 0 to 10). Sliding scales were used for the pain severity and pain interference items; the number of pain attacks and continuous pain duration were entered by typing in. The pain severity scales included smiley faces and short verbal descriptions to help participants contextualize their response. Participants could neither edit nor review their previous entries.

#### 2.4.3 Interview procedure

Participants took part in a qualitative, semi-structured interview within 4 weeks of completing the TnED©. All interviews from the UK site were conducted virtually (via Microsoft Teams App) by SAD and SC who were initially supervised by one of the study authors (JMZ). The interviews at the US site were conducted using either web-assisted technology (*i.e.*, Microsoft Teams or Zoom App) or face-to-face. To ensure consistency, all interviews were conducted by the same study coordinator.

All interviews were conducted using a semi-structured discussion guide. Both sites explored the content of the items that make up the TnED©; the UK site team asked additional questions which explored the acceptability of long-term use of the instrument and alternative uses (*e.g.*, as a communication tool between the patient and treating physician outside a clinical trial setting). Overall, there were some brief initial questions designed to establish rapport and open the conversation. This step of concept elicitation (CE) allowed the research team to collect the symptoms and impact of TN, as spontaneously reported by participants. The main purpose of CE was to evaluate whether all reported concepts were covered by the questions asked in the TnED©. CE was followed by a cognitive debrief (CD), a series of focused questions to debrief the items that make up the TnED©, which aimed at assessing participants’ understanding of the instructions and items, as well as item relevance and the suitability of the recall period and response options.

All interviews were audio-recorded and transcribed verbatim for analysis. Interview transcripts were reviewed by the lead researcher (JMZ, HA) at each site.

Example questions from the discussion guides can be found in Fig. 1.

**Fig. 1.** Examples of interview questions.

### 2.5 Analytic methods

Participant demographic data were summarized to characterize the sample and provide context to the qualitative data.

#### 2.5.1 Qualitative analysis

All transcripts were reviewed to verify accuracy and remove identifiable information. Following this, the de-identified, pseudo-anonymized transcripts were uploaded to the software NVivo (version 14, Ritme, Paris, France), a program that assists in the coding of qualitative data and allows for codes to be assigned to portions of text. These codes can then be combined and grouped with other codes as needed such that relationships can be identified.

#### 2.5.2 Analysis of concept elicitation data

The CE analysis aims to detect “concepts of interest” for patients, meaning relevant key symptoms, and disease impacts relevant to be measured with the instrument.

Analysis of CE data began with an exploratory session where the pseudo-anonymized transcripts from the UK interviews were analyzed by the UK lead researcher, sponsor representative, and 2 researchers. The content of this first exploratory analysis was then shared with the US research team.

After this exploratory session, comprehensive thematic analysis of qualitative data was conducted on the full data by 2 expert researchers from the independent vendor specialized in PROM validation. The aim of the thematic analysis was to extract meaning from the interview text, allowing flexibility in linking the content of the interviews to the theoretical understanding of TN. There were 6 main steps to the thematic analysis: familiarization, generation of codes (concepts), identification of themes, review and refinement of themes, and summarization of codes and themes.

#### 2.5.3 Analysis of cognitive debriefing data

Cognitive Debriefing (CD) consists of a thematic analysis of participant answers to questions on the clarity/ comprehension of the questions composing the instrument, recall period, response options, and feedback related to missing/additional items or reformulation suggestions to increase clarity. Analysis of CD data focused on quotes pertaining to the main research questions, including relevance, suitability, comprehension, and any rewording suggestions for TnED© items. CD data were analyzed using structured, deductive coding that systematically compares participant responses. Any suggestions to improve the instructions or items of the measure were also coded.

## 3. Results

### 3.1 Demographic characteristics

A total of 30 participants with a confirmed diagnosis of TN took part in the interviews to provide feedback on the TnED© items; demographic data are presented in Table 1. More female participants (80%; n = 24) than males (20%; n = 6) were interviewed. Participants were between 30 and 75 years, with a mean age of 54.9 and a standard deviation (SD) of ±15.17 (n = 23; age data not recorded for 7 participants). Fifty percent of the sample had a diagnosis of TNP while the remaining half had TNC. The mean age and gender split was consistent across TN types.

**Table 1.** Demographic data of study participants.

Demographic Variables		Total (N = 30)	TNP (n = 15)	TNC (n = 15)
Age (yr)				
	Mean (standard deviation)	54.9 (15.17)	56.5 (14.86)	53.4 (15.95)
	Median	60	62	52
	Min, Max	30, 75	33, 72	30, 75
	Missing*	7	4	3
Gender, n (%)				
	Female	24 (80%)	11 (73%)	13 (87%)
	Male	6 (20%)	4 (27%)	2 (13%)

Max: maximum value; Min: minimum value; TNC: trigeminal neuralgia with concomitant continuous pain; TNP: trigeminal neuralgia with purely paroxysmal pain. *Demographics were not consistently collected by sites and thus some missing data are present.

### 3.2 Key findings of cognitive debrief of TnED© items

Participants were able to complete the instrument daily, but some participants missed entries for a variety of reasons. During CD, nearly all participants stated that the items were clear and easy to understand, relevant, and had a suitable recall period and response options (see Table 2). Only 1 participant with TNC reported struggling with the understanding of 1 particular item (*i.e.*, number of pain attacks).

**Table 2.** Overall frequency counts for the cognitive debrief of the TnED©.

Item number	Response	Item understanding	Item relevance	Suitable response options	Suitable recall period
TNP (n = 15)					
1 Number of pain attacks					
	Yes	14	15	7*	15
	No	0	0	0	0
	Missing	1	0	8	0
2 Pain attack severity					
	Yes	14	15	7	15
	No	0	0	0	0
	Missing	1	0	8	0
3 Interference with daily activities					
	Yes	14	15	7	15
	No	0	0	0	0
	Missing	1	0	8	0
TNC (n = 15)					
1 Number of pain attacks					
	Yes	14	15	12	15
	No	1	0	0	0
	Missing	0	0	3	0
2 Pain attack severity					
	Yes	15	15	12	15
	No	0	0	0	0
	Missing	0	0	3	0
3 Continuous pain duration					
	Yes	15	15	12^†^	15
	No	0	0	0	0
	Missing	0	0	3	0
4 Continuous pain severity					
	Yes	15	15	12	15
	No	0	0	0	0
	Missing	0	0	3	0
5 Interference with daily activities					
	Yes	15	15	12	15
	No	0	0	0	0
	Missing	0	0	3	0

TNC: trigeminal neuralgia with concomitant continuous pain; TNP: trigeminal neuralgia with purely paroxysmal pain. *Three out of 7 participants said the response options were suitable, although quantifying the number of TN attacks accurately may be challenging. ^†^Seven out of 12 participants said the response options were suitable; however, quantifying the number of TN attacks and continuous pain duration accurately may be challenging.

Although minor modifications were suggested by some participants, these did not impact participants’ ability to understand or complete the items in the TnED©. Some changes were more frequently discussed by participants, while others can be considered idiosyncratic.

#### 3.2.1 Item understanding and relevance

For participants with TNP, 14/15 participants (1 participant with missing data) indicated that all items were clear and easy to understand: “*Yes it was easy to complete and I understood it very well.*” (Participant 01-1008, TNP). For participants with TNC, 14/15 participants indicated that the items cognitively separated continuous pain from pain during paroxysms, and only 1 participant reported that an item (Number of pain attacks) was confusing in this regard.

All participants reported that all TnED© items were relevant to their experience of TN.

##### 3.2.1.1 Most important concept: paroxysmal pain severity *vs.* paroxysmal pain frequency *vs.* presence of continuous pain (US site only)

In addition, of the 12 participants from the US asked to identify the most important concept to them, 83.3% (10/12) reported severity of flare-ups (paroxysms), 16.7% (2/12) reported presence of continuous pain, and none reported frequency of flare-ups.

##### 3.2.1.2 Most important item (UK site only)

Furthermore, 18 participants from the UK were asked to identify which item was the most important to them:

● Pain interference item: 44.4% (8/18) participants.

● Pain interference, frequency, and severity items are all equally important: 27.8% (5/18) participants.

● Pain frequency item: 11.1% (2/18) participants.

● Pain severity item: 5.6% (1/18) participants.

● No clear answer: 11.1% (2/18) participants.

The pain interference item was most frequently cited as being the most important as it captured the impact of the participants’ TN-related face pain on their daily activities: “*… it has affected my quality of life substantially but I never noted that down. And it made me really realize how over the many, many 23 years, how it has affected me in everything I do …*” (Participant identification code (ID): 002-012).

#### 3.2.2 Response options for the TnED©

All 19 participants (TNP: n = 7; TNC: n = 12) who discussed the TnED© response options reported that these were suitable (19/19, 100%) (see Table 2).

Participants also stated that the smiley face design of the response options for items measuring pain attack severity was helpful.

Despite all participants stating that the response options were appropriate, 10 participants (TNP: n = 3/7; TNC: n = 7/12) provided some feedback on the ease of responding to the items:

● 7 participants described how for item 1 (number of pain attacks), they felt that sometimes it can be hard to accurately remember the exact number of paroxysms they had experienced when the paroxysm frequency was high.

● 10 participants stated that for item 1 (number of pain attacks), the different sub-periods throughout the day (morning, midday, evening, night), while sometimes being helpful, could also become challenging when compared with providing a score for the whole day, because the sub-periods were not defined. In some cases, the ambiguity regarding the definition of sub-periods led to some attacks not being recorded: “*So there maybe were more attacks that were happening in the evening that weren’t being documented.*” (Participant ID: 01-001, TNC). The decision not to define the sub-periods (*e.g.*, define morning as 6:00–10:00 AM) was intentional in the design of the instrument. Since the instrument analyzes only daily aggregate data, day sub-periods were left undefined to provide flexibility to each participant to define these time periods according to their own daily routine.

● 4 participants with TNC discussed how sometimes it can be hard to calculate the time spent in continuous pain.

#### 3.2.3 Recall period for the TnED©

All participants (N = 30) were able to recall the information about their TN-related face pain over the past 24 hours for all items, meaning that the recall period was suitable (see Table 2). Several participants reported missing data entries because they forgot to log their symptoms or because of planned activities, and therefore suggested a longer window to complete data.

One participant reported that a recall period longer than 1 day would make it difficult to remember the pain and could lead to less accurate data.

#### 3.2.4 Suggested changes to the TnED©

Some participants suggested minor changes they felt would help improve the measure. None of the suggested changes, however, impacted participants’ ability to complete the measure.

The main changes suggested by participants revolved around providing more information about their attacks, as they felt this might help to explain the reported data. These suggestions included adding a free text box, or questions regarding what triggered their pain attack.

All suggested changes are presented in Table 3, along with feedback regarding whether each suggestion is retained for implementation.

**Table 3.** Suggestions for change to the TnED© items across both TN phenotypes.

Suggestion	n	Example Quote(s)	Potential Future Changes
Include a comment box to collect additional information about answers	14	“*The only thing I would have kind of added would be a note section, just because if you had, well you could have had some really good days and then you might have had a ton of pain on one particular day but you might know exactly what triggered it.*” (Participant ID: 02-011, TNP)	If this is information that the research team finds useful, this could be collected via alternative measures.
Include a comment box where the triggers for the attacks can be listed	13	“*Maybe that’s something then you can track and maybe triggers, were there certain triggers throughout the day as well? And maybe at the end of the month you would realize what different triggers were there that you didn’t realize. Because it’s hard to know what triggers are there at the time, but maybe at the end of a couple of weeks you’ll maybe pick up a pattern, you know.*” (Participant ID: 02-001, TNP)	If this is information that the research team finds useful, this could be collected via alternative measures.
Clarify time periods in item 1 response options	10	“*I found it (item 1 time periods) quite confusing in that respect just from the time periods, you know, but I mean if it was through the night I kind of classed that as morning really.*” (Participant ID: 02-004, TNC)	Daily time periods are included to aid the participants and are grouped when scoring each item. Therefore, this is not required as data is not scored to this level of granularity.
Differentiate between pain type	10	“*I would go with the episodic or electrical pain vs the continuous pain. In fact, it would really clarify if you put an instruction in the beginning, that said, and people would only read it once, but it would show up every day- We’re interested in two kinds of pain. You may or may not have both. We’ll be asking about electrical pain, shooting pain, and then we’re going to ask about continuous pain. Because then people can make that distinction. For people that have both.*” (Participant ID: 01-004, TNC)	Participant descriptions of pain varied hence no consistent language was suggested. This is subjective and is covered as part of instrument (diary) training.
Show history	9	“*…. But I couldn’t go back and look at all my other entries. If I was using it, yeah I think I’d like to look back. Especially if I’d started a new medication, to look back and see how it changed then that would be really handy. But, you know, I did enjoy using it.*” (Participant ID: 02-014, TNC)	This would be useful if the instrument were being used to help inform the health care provider about the impact of different medications and dosage.
Include impact (mental health, ADLs *etc.*)	8	“*…I would like to see something included regarding mood on a mental health side or a portion of how Trigeminal Neuralgia affects mood, …*” (Participant ID: 01-001, TNC)	In clinical practice important to capture but probably not on a daily basis.
Include association of pain with medication use	6	“*Because for me it was hard because I’m changing medication every week to try and stop a major attack. So mine’s getting worse and worse at the moment with a shift. And then one day I’ll take a new medication and perhaps it effects a bit. So the numbers were constant against the shifting picture so I don’t know whether having a picture would either make it blurred or would make it more valuable.*” (Participant ID: 02-016, TNP)	This would be useful if the instrument is being used to help inform the health care provider about the impact of different medications and dosage.
Additional symptoms	2	“*…. so that people could define their own symptoms and then you still have the chart if someone does not have additional symptoms.*” (Participant ID: 02-018, TNP)	This could be part of comments but would not be needed on a daily basis.

*TNC: trigeminal neuralgia with concomitant continuous pain; TNP: trigeminal neuralgia with purely paroxysmal pain; ID: identification code.*

### 3.3 Key findings of concept elicitation

All concepts reported by participants during the CE phase as being important were addressed in the questions which comprise the TnED©.

### 3.4 Usability of the app presenting TnED© items

As part of the interviews, some participants also commented on the usability of the items in the TnED©.

#### 3.4.1 Ease of use

Nearly all participants (27/29, 93.1%) reported that the TnED© was easy to use. In addition, the participants described how they liked the simplicity of the app and the design of the items: “*I liked that it was quite a simple interface. The sliding scale is very easy to use, all of it was really clear. The ‘next’ button is nice and clear so I thought it had been well designed for its purpose.*” (Participant ID: 02-017, TNC). The rest of the participants (2/29, 6.9%) described how, on a couple of occasions, they had technical issues with the App, which caused frustration.

#### 3.4.2 Reminders

Participants described how the email reminders which prompted evening completion of the TnED© and highlighted missed entries were useful, with some participants also setting reminder alarms: “*I used it every day. I set myself an alarm to prompt me to do it but then I also got the emails to remind me.*” (Participant ID: 02-014, TNC). In some instances, participants reported receiving multiple reminders but reiterated that this was still helpful, and did not bother them.

#### 3.4.3 Time for completion

All 19 participants (TNP: n = 7; TNC: n = 12) who discussed the TnED© response options said they found the TnED© items quick to complete, taking 5 minutes or less, and described them as having a low burden. In addition, participants described how time for completion shortened as they became accustomed to the process.

#### 3.4.4 Usability feedback

##### 3.4.4.1 Electronic device type

Overall, most participants found the electronic format of the TnED© easy and convenient.

There were mixed reviews of the sliding scale, with 3 participants providing appreciative feedback, and 5 participants reporting difficulty using the slider or entering-in a response.

A larger device was proposed by 2 participants to enhance usability.

##### 3.4.4.2 Allocated completion periods

Of the 25 participants who discussed the allocated time for completion of the items in the TnED© (every evening), all (25/25, 100%) found this to be suitable.

##### 3.4.4.3 Length of administration period

Of the 26 participants who were asked about the 2-week completion period for the TnED©, all (26/26, 100%) found this suitable. However, 3 participants suggested that 2 weeks did not fully capture the fluctuations in pain that may occur. Of these 3 participants, 2 suggested that 4 weeks would provide a more accurate representation of their pain experience.

When asked if they could complete the TnED© over a longer period, for example longer than 20 weeks as part of a clinical trial, all participants (26/26, 100%) expressed that they envisaged this would be easy to do, mainly due to the simplicity of the TnED©.

## 4. Discussion

The TnED© is the first electronic instrument for TN pain which covers severity, frequency of pain attacks, duration and severity of continuous pain, as well as impact on QoL. The study evaluated both patients with TNP and patients with TNC. Positive feedback on the items that make up the TnED© was consistent regardless of the TN phenotype or the research site country. The sample size of 30 participants provided sufficient feedback to evaluate the TnED© for future use.

Content validity, conceptualized as the degree to which the content of a PROM is an adequate reflection of the construct to be measured, is considered the most important measurement property, because the items of the PROM should be relevant, comprehensive, and comprehensible with respect to the construct of interest and study population [13]. All 5 items (3 shared items for TNP and TNC, and 2 additional items for TNC) of the TnED© received a maximal relevance score from participants. Regarding comprehensiveness, all concepts reported by participants during the CE phase as being important (pain severity, number of pain attacks, *etc.*) are measured by the instrument. Nineteen participants, who discussed the response options, felt that these were suitable. Some participants suggested additional items to be collected, including: (1) information about pain triggers and concomitant pain medication; (2) pain location; (3) effect on mood/mental health; and (4) patient-defined symptoms. Recently, different TN stakeholders (patients, clinicians, and researchers) identified a Core Outcome Set (*i.e.*, a disease-specific minimum data set to be collected and reported in clinical trials) for trigeminal neuralgia (TRINCOS), with consensus reached on 10 outcomes across six domains (pain, side effects, social impact, quality of life, global improvement, and satisfaction with treatment) [10]. Three of the 10 outcomes that met TRINCOS study criteria are also featured in the TnED©, namely pain intensity, pain interference, and the ability to participate in social roles and activities. Interestingly, the impact of treatment on mood, specifically anxiety and depression in the TRINCOS study, failed to meet study criteria in TRINCOS, despite the understanding that TN pain affects mental well-being [4]. Regarding item clarity, 14 out of 15 participants (1 missing participant with TNP, and 1 TNC participant who replied “No” to the “Number of pain attacks” item) in each diary group indicated they understood each of the items of the TnED©.

Ease of use is a key factor influencing the intention to continue using a mobile application [14]. In the present study, the TnED© was reported as being easy to use by 27 out of 29 participants, and 26 out of 26 participants expressed that it would be easy to complete the TnED© for longer (*e.g.*, more than 20 weeks). The small number of questions, taking less than 5 minutes to complete, was a commonly cited reason for this impression. The smiley faces were well received by participants, in line with previous studies in both pediatric and adult populations [15, 16]. However, participants expressed some concerns related to the ease of use of the TnED©. Some indicated it was hard to accurately remember the exact number of pain attacks (paroxysms) they had experienced when paroxysm frequency was high. The sliding scale, used to select scores from 0 to 10, received mixed reviews, with 5 participants reporting difficulty using it. The 24-hour recall period was regarded as suitable by every participant; however, 4 participants indicated that some of their pain-related information was not captured because the reporting window was closed.

A possible future use of the TnED© could be to aid TN healthcare professionals with trend information on changes in TN symptoms, triggers, and medication effectiveness. Interest in this was expressed by several participants. Artificial intelligence could be used to extract this information if comment boxes or an additional text field were incorporated into the TnED© in the future [17].

### 4.1 Strengths

Strengths of this study include the novelty of evaluating an electronic instrument for TN pain, the large sample size, the use of a multidisciplinary team to develop the instrument, selection of items in the TnED© that agree with the CE results, and consistency with some of the most important domains identified in the TRINCOS study [10].

### 4.2 Limitations

All participants were English-speaking from the UK and the US and the concepts captured by the TnED© items appear to reflect the general lived experience of patients with TN. However, if the TnED© is translated into other languages, appropriate cultural and linguistic validation should be conducted in accordance with best practices for PROM translation [18]. More female participants were recruited than males, consistent with TN prevalence patterns [19]. To reduce participant burden and increase geographic diversity (recruit patients living farther from the study centers), interviews were conducted using both in-person and virtual methods. While a single method would have been preferable for consistency, no differences in the data collected were observed between these two methods. Similarly, it would have been preferable to use a single interviewer; however, the UK site used 2 interviewers, while the US site used one interviewer. To ensure consistency, the US interviews were supervised by a single research team member, and a supervisor was present for the initial UK interviews. Importantly, no patterns in the data related to the main research questions suggested that interviewer number affected the data obtained.

## 5. Conclusions

Overall, this study has provided evidence in support of the content validity of the TnED©. The TnED© items capture pain dimensions relevant to patients with both TN phenotypes. Nearly all participants found the TnED© items clear, easy to understand, and relevant, with suitable response options and recall period. Participants consistently reported that the instrument is easy to use and quick to complete, and that the completion time was appropriate and the email reminders were helpful. All participants indicated they would be able to complete the TnED© for durations typical of clinical trials. Few changes to the instrument were suggested, and after careful consideration, the study sponsor decided not to implement them. The electronic format was deemed convenient and appeared to reduce the burden associated with paper-based diaries.

The TnED© has the potential to aid tailored treatment approaches for TN patients and provide clinicians with comprehensive data to support the holistic management of TN. Validation of the psychometric properties of the TnED© is ongoing.
